# Phylogenomic Approaches to DNA Barcoding of Herbal Medicines: Developing Clade-Specific Diagnostic Characters for *Berberis*

**DOI:** 10.3389/fpls.2019.00586

**Published:** 2019-05-14

**Authors:** Marco Kreuzer, Caroline Howard, Bhaskar Adhikari, Colin A. Pendry, Julie A. Hawkins

**Affiliations:** ^1^School of Biological Sciences, University of Reading, Reading, United Kingdom; ^2^BP-NIBSC Herbal Laboratory, National Institute for Biological Standards and Control, Potters Bar, United Kingdom; ^3^Royal Botanic Garden Edinburgh, Edinburgh, United Kingdom

**Keywords:** DNA barcoding, next-generation sequencing, operational phylogenetic units, herbal medicines, *Berberis*, pharmacopoeia, pharmacopoeial standards, plastome

## Abstract

DNA barcoding of herbal medicines has been mainly concerned with authentication of products in trade and has raised awareness of species substitution and adulteration. More recently DNA barcodes have been included in pharmacopoeias, providing tools for regulatory purposes. The commonly used DNA barcoding regions in plants often fail to resolve identification to species level. This can be especially challenging in evolutionarily complex groups where incipient or reticulate speciation is ongoing. In this study, we take a phylogenomic approach, analyzing whole plastid sequences from the evolutionarily complex genus *Berberis* in order to develop DNA barcodes for the medicinally important species *Berberis aristata*. The phylogeny reconstructed from an alignment of ∼160 kbp of chloroplast DNA for 57 species reveals that the pharmacopoeial species in question is polyphyletic, complicating development of a species-specific DNA barcode. Instead we propose a DNA barcode that is clade specific, using our phylogeny to define Operational Phylogenetic Units (OPUs). The plastid alignment is then reduced to small, informative DNA regions including nucleotides diagnostic for these OPUs. These DNA barcodes were tested on commercial samples, and shown to discriminate plants in trade and therefore to meet the requirement of a pharmacopoeial standard. The proposed method provides an innovative approach for inferring DNA barcodes for evolutionarily complex groups for regulatory purposes and quality control.

## Introduction

DNA barcoding has two major objectives: specimen identification, where an unknown sequence is matched to a sequence of a known species, and species discovery, which is equivalent to species delimitation and species description (DeSalle, 2006). DNA barcoding of herbal medicines is mainly concerned with authentication, the identification of specimens for quality assurance (Sgamma et al., 2017). In the last decade, DNA barcoding of herbal medicines has raised awareness of species substitution and adulteration, highlighting issues surrounding the quality of herbal medicines in the global market (Newmaster et al., 2013; Srirama et al., 2017). Regulation of herbal medicines is a pressing issue for regulatory agencies (Directive 2001/83/Ec, 2001; Directive 2004/83/EC, 2004; Vlietinck et al., 2009). Published pharmacopoeial standards for authentication predominantly rely on chemical and anatomical methods (e.g., British Pharmacopoeia, 2016), but DNA barcoding offers new tools for regulatory purposes (de Boer et al., 2015) and DNA barcodes have recently been incorporated into the British Pharmacopoeia for the first time (British Pharmacopoeia Commission, 2017). Here we investigate opportunities and limitations of DNA barcoding using next-generation sequence data of an evolutionarily complex genus. The aim is to design new methodological approaches for producing DNA barcodes for regulatory purposes, pharmacovigilance and quality assurance.

To date, the British Pharmacopoeia has approved 6 annotated DNA barcodes for the individual identification of the following species: *Anethum graveolens* Sowa (ITS2); *Glehnia littoralis* (ITS2); *Ocimum tenuiflorum* (*trnH-psbA*); *Myristica fragrans* (*trnH-psbA*); *Phellodendron amurense* (*trnH-psbA*); and *Phellodendron chinense* (*trnH-psbA*). The British Pharmacopoeia Commission (2017) have also published guidelines for the use of these barcodes, guiding users through the extraction of DNA, amplification of barcode markers, sequencing and comparison to pharmacopoeial standards. This development of bespoke barcode markers for different species is an approach likely to continue since there is no single, universal DNA barcode for land plants (Hollingsworth et al., 2011). For taxonomic purposes, several propositions have been made (e.g., Kress et al., 2005; Chase et al., 2007; CBOL Plant Working Group et al., 2009). Following Hollingsworth et al. (2011), most studies use a combination of the plastid regions *matK, rbcL*, the intergenic spacer *trnH*-*psbA* and the nuclear ITS2. Advances in sequencing technology have encouraged the barcoding community to augment the standard barcoding approach (Kane et al., 2012; Vaughn et al., 2014; Coissac et al., 2016; Zhang et al., 2017). In the era of next-generation sequencing, some researchers have even argued for the use of whole plastid genomes as barcodes (Kane et al., 2012; Vaughn et al., 2014; Coissac et al., 2016; Zhang et al., 2017; Manzanilla et al., 2018). How whole plastid genomes might be best deployed for pharmacopoeial purposes has hardly been explored yet.

Methodological approaches for specimen identification using DNA barcodes commonly rely on either distance-based measures or phylogenetic methods (Austerlitz et al., 2009). The former are based on the assumption that intra- and interspecific variation do not overlap (e.g., Hebert et al., 2004), also referred to as the barcoding gap (Meyer and Paulay, 2005). Accurate specimen identification using distance-based approaches such as BLAST are highly dependent on a well-curated database in which all members of a group are ideally represented by several individuals (Meyer and Paulay, 2005). The drawbacks of using distance-based approaches are that there is no objective distance threshold criterion and that the nearest neighbor is not always the closest relative (Moritz and Cicero, 2004). Specimen identification using phylogenetic methods is based on membership of a query sequence to a specific clade (Casiraghi et al., 2010). One difficulty associated with using tree-based barcoding methods is that phylogenies inferred from the barcode sequence might not be resolved sufficiently for an individual to be allocated to a clade, and that clades may exhibit poor support, questioning the robustness of any phylogenetic hypothesis (Moritz and Cicero, 2004). The use of concatenated DNA sequences for species tree inference has been shown to produce more robust phylogenetic hypotheses (Rokas et al., 2003). However, phylogenetic methods of DNA barcoding are not suitable when the underlying system is not based on strictly hierarchical ancestor-descendant relations structures, such as in nested structures (Goldstein and DeSalle, 2005).

Whether specimens of different species can be differentiated depends on the choice of the DNA barcode and the reproductive isolation and evolutionary history of the species under investigation. Although relatively high success rates for the identification of genera has been reported when using common barcodes in plants, limited sequence variation is often the cause of the failure to distinguish between closely related species (Seberg and Petersen, 2009; Parmentier et al., 2013; Braukmann et al., 2017). One incentive for employing genomic approaches for barcoding is that broader genome coverage increases the variation in the barcoding data set (Coissac et al., 2016). However, closely related species may not exhibit a DNA barcoding gap even when the most variable regions are employed. In the case of incipient speciation where lineage sorting is incomplete, species are likely to be paraphyletic (Rieseberg and Brouillet, 1994; Fazekas et al., 2009). Furthermore, cytoplasmic genomes can have different evolutionary histories compared with nuclear genomes because of processes such as chloroplast capture (Rieseberg and Soltis, 1991), and specimens may group geographically rather than taxonomically (Acosta and Premoli, 2010). The success of DNA barcoding may therefore be limited in some plant groups because of their biology and evolutionary history (Percy et al., 2014).

The genus *Berberis* is a case in which DNA barcoding using only a few regions has had limited success (Roy et al., 2010). Similarly, a phylogeny of *Berberis* based on *ndhF* and ITS loci failed to resolve boundaries of several species (Adhikari et al., 2015). *Berberis aristata* is a medicinal plant that has been in traditional use in India for centuries and is nowadays traded throughout the world (Srirama et al., 2017). Local market studies suggest that several species are traded under the same vernacular name (Srivastava and Rawat, 2013), including *B. aristata* and *B. asiatica. B. aristata* is described in several pharmacopoeias (Ayurvedic Pharmacopoeia of India, 2001; British Pharmacopoeia, 2016). Chemical and anatomical tests are deficient and conventional macro-morphological and microscopic examination do not distinguish the traded materials (Chandra and Purohit, 1980; Srivastava et al., 2004) therefore there is a strong incentive for the development of a DNA barcoding method for their identification.

The aim of this study is to investigate whole plastid sequences of the genus *Berberis* as a resource for barcode design, utilizing a whole plastid phylogeny of the species in order to better understand the difficulties of using barcoding for pharmacopoeial purposes. In light of the challenges of this complex group, we develop a method for identifying short, informative plastid barcode regions based on diagnostic nucleotides. These barcodes, which are informative of clade membership in a phylogenetic context, are tested on commercial samples, and their utility for regulatory purposes and quality control outlined.

## Materials and Methods

### Sampling

This study includes 85 specimens from 57 species (Table 1). The dataset includes sequences from two putative new species (named in this study as B_newsppA and B_newsppB) and one unidentified species (B_spp).

**Table 1:** Specimen information.

Sample	Species	Locality	Lat.	Long	Collector(s)	Coll. Date	Voucher	Comments
B_angulosa1	*B. angulosa* Wall. ex Hook.f. & Thomson	Nepal, Illam District	27.11	87.99	Adhikari, B. et al.	14-Jun-07	LKSRB71	
B_angulosa2	*B. angulosa* Wall. ex Hook.f. & Thomson	Nepal, Rasuwa District	28.21	85.57	Adhikari, B.	03-Aug-07	BL244	
B_angulosa3	*B. angulosa* Wall. ex Hook.f. & Thomson	Bhutan, Haa	27.27	89.17	Di McNab	01-Jul-05	AS97	Cultivated (J. Harber Coll.)
B_aristata10	*Berberis aristata* DC.	Nepal, Dhankuta District	27.04918	87.35425	Adhikari, B. et al.	01-Aug-14	WP21.1	
B_aristata11	*Berberis aristata* DC.	Nepal, Gandaki District	28.39255	83.77315	Adhikari, B.	05-Oct-06	EA109	
B_aristata3	*B. aristata* DC.	Nepal, Dhankuta District	27.05	87.35	Adhikari, B. et al.	01-Sep-14	WP21.5	
B_aristata4	*B. aristata* DC.	N/A	N/A	N/A	N/A	N/A	1260210	
B_aristata6	*Berberis aristata* DC.	Nepal, Koshii District	27.04918	87.35425	Adhikari, B. et al.	01-Aug-14	WP32.5	
B_aristata7	*Berberis aristata* DC.	Nepal, Koshii District	27.04048	87.31713	Adhikari, B. et al.	01-Aug-14	WP18.2	
B_aristata8	*Berberis aristata* DC.	Nepal, Dhawalagiri District	28.66222	83.59472	Adhikari, B.	17-Aug-07	EA243	
B_aristata9	*Berberis aristata* DC.	Nepal, Dhawalagiri District	28.66028	83.59389	Adhikari, B.	17-Aug-07	EA249	
B_asiatica2	*B. asiatica* Roxb. ex DC.	Nepal, Makwanpur District	27.58	85.16	Adhikari, B. et al.	25-Aug-17	Coll_7.1	
B_asiatica4	*B. asiatica* Roxb. ex DC.	India, no further details	N/A	N/A	C. Chadwell	N/A	AS82	Cultivated (J. Harber Coll.)
B_asiatica5	*Berberis asiatica* Roxb. ex DC.	Nepal, Narayani Zone	27.6541	85.09973	Adhikari, B. et al.	01-Aug-14	Coll_38.1	
B_asiatica6	*Berberis asiatica* Roxb. ex DC.	Nepal, Bagmati Zone	27.77278	85.43166	Adhikari, B. et al.	02-Sep-14	SB1	
B_calliantha	*B. calliantha* Mulligan	China, Tibet	28.91	89.61	F. Kingdon-Ward, Ex Hillier	21-Nov-24	AS38	Cultivated (J. Harber Coll.)
B_chrysosphaera	*B. chrysosphaera* Mulligan	China, Tibet	28.65	97.46	F. Kingdon-Ward, Ex Hillier	10-Dec-33	AS39	Cultivated (J. Harber Coll.)
B_con_extensiflora1	*B. concinna* var. *extensiflora* Ahrendt	Nepal, Manang District	28.61	84.47	N/A	14-Aug-08	20812277	
B_con_extensiflora2	*B. concinna* var. *extensiflora* Ahrendt	Nepal, Myagdi District	28.4	83.69	N/A	04-Oct-06	EA104	
B_con_extensiflora3	*B. concinna* var. *extensiflora* Ahrendt	Nepal	N/A	N/A	C. Chadwell	N/A	AS74	Cultivated (J. Harber Coll.)
B_concinna2	*Berberis concinna* Hook.f.	India, Sikkim	27.83472	88.69944	T D. Atkinson	05-Jul-05	AS102	
B_concolor	*B. concolor* W. W. Smith	China, Yunnan	28.47	98.91	D. E. Boufford et al.	20-Aug-13	43135	
B_congestiflora	*B. congestiflora* Gay	Chile, Región IX	N/A	N/A	Gardner et al.	19-Feb-88	1988.0916	Cultivated (RBGE)
B_cooperi	*B. cooperi* Ahrendt	Bhutan, Timphu	27.47	89.64	J. F. Harber s.n.	01-Aug-97	AS9	Cultivated (J. Harber Coll.)
B_crassilamba	*B. crassilamba* C. Y. Wu ex S. Y. Bao	China, Yunnan	27.61	99.89	D. E. Boufford et al.	04-Sep-13	43437	
B_darwinii	*B. darwinii* Hook.	Argentina : Prov. Río Negro	N/A	N/A	Unknown	N/A	1987.2408	Cultivated (RBGE)
B_derogensis	*B. derogensis* T. S. Ying	China, Sichuan	29.09	99.38	D. E. Boufford et al.	22-Aug-13	43164	
B_dictyophylla1	*B. dictyophylla* Franch.	China, Yunnan	27.89	99.68	B & S Wynn-Jones	17-Sep-00	AS93	Cultivated (J. Harber Coll.)
B_dictyophylla2	*B. dictyophylla* Franch.	China, Yunnan	25.94	100.4	Z. W. Liu s.n.	N/A	AS100	Cultivated (J. Harber Coll.)
B_everestiana1	*B. everestiana* var. *ventosa* Ahrendt	Nepal, Solu Khumbu District	27.86	86.64	N/A	23-Sep-05	DNEP3BY156	
B_everestiana2	*B. koehneana* C. K. Schneid.	Nepal, Mustang District	28.82	83.86	Adhikari, B.	16-Aug-07	EA217	
B_fendleri	*B. fendleri* A.Gray	N/A	N/A	N/A	N/A	N/A	N/A_2	Cultivated (RBGE)
B_glaucocarpa	*B. glaucocarpa* Stapf	Nepal, Doti District	29.35	81.06	N/A	01-Jul-09	20918011	
B_graminea	*B. graminea* Ahrendt	China, Sichuan	28.12	101.18	D. E. Boufford et al.	06-Sep-13	43466	
B_griffithiana1	*B. griffithiana* C.K.Schneid.	India, Arunachal Pradesh	27.58	91.88	SF 06008	24-Nov-06	AS55	Cultivated (J. Harber Coll.)
B_griffithiana2	*B. griffithiana* C.K.Schneid.	India, Arunachal Pradesh	27.33	92.31	A Clark 5260	01-Oct-04	AS54	Cultivated (J. Harber Coll.)
B_grodtmanniana	*B. grodtmanniana* C. K. Schneider	China, Sichuan	27.69	101.22	D. E. Boufford et al.	06-Sep-13	43471	
B_gyalaica1	*Berberis gyalaica* Ahrendt ex F.Br.	China, Tibet	29.65056	94.36	W. Bentall	27-Jun-05	WB	
B_gyalaica2	*Berberis gyalaica* Ahrendt ex F.Br.	China, Tibet	28.97444	93.69472	W. Bentall	NA	AS6	Cultivated (J. Harber Coll.)
B_hamiltoniana	*Berberis hamiltoniana* Ahrendt	Nepal, Bajhang District	29.61553	81.00556	Adhikari, B.	NA	20915095	
B_hookeri2	*B. hookeri* Lem.	Nepal, Khumbu District	27.76	86.71	N/A	29-Sep-05	DNEP3BY213	
B_hookeri5	*Berberis wallichiana* DC.	Nepal, Panchthar District	27.10263	87.96897	Adhikari, B. et al.	08-Jun-07	LKSRB28	
B_hookeri6	*Berberis hookeri* Lem.	Nepal, Myagdi District	28.4014	83.70257	Adhikari, B.	04-Oct-06	EA106	
B_hookeri7	*Berberis hookeri* Lem.	Nepal, Myagdi District	28.40443	83.69923	Adhikari, B.	13 July 2009	Bajhang0920915095	
B_insignis	*Berberis insignis* Hook.f. & Thomson	Nepal, Illam District	27.06317	88.01702	Adhikari, B. et al.	16-Jun-07	LKSRB144	
B_jaeschkeana1	*B. jaeschkeana* var. *usteriana* C.K.Schneid.	Nepal, Jumla District	29.32	82.18	N/A	03-Jun-08	JRSA12	
B_jaeschkeana2	*Berberis jaeschkeana* var. *usteriana* C.K.Schneid.	Nepal, Mustang District	28.71222	83.55889	Adhikari, B.	17-Aug-07	EA238	
B_jamesiana2	*B. jamesiana* Forrest & W. W. Smith	China, Yunnan	26.11	100.17	D. E. Boufford et al.	14-Sep-13	43530	
B_karnalensis	*B. karnaliensis* Bh.Adhikari	Nepal, Jumla District	29.3	82.18	N/A	03-Jun-08	JRSA5	
B_koehneana	*B. koehneana* C. K. Schneid.	Nepal, Mustang District	28.68	83.6	N/A	30-Sep-06	EA56	
B_kumaonensis	*B. kumaonensis* C. K. Schneid.	Nepal, Doti District	29.38	81.12	N/A	02-Jul-09	20915029	
B_leptopoda	*B. leptopoda* Ahrendt	India, Arunachal Pradesh	28.57	95.06	K. Rushforth		AS103	Cultivated (J. Harber Coll.)
B_levis	*B. levis* Franch.	China, Yunnan	25.96	100.39	D. E. Boufford et al.	15-Sep-13	43557	
B_mekongensis	*B. mekongensis* W. W. Smith	China, Yunnan	28.33	99.12	D. E. Boufford et al.	19-Aug-13	43131	
B_micropetala	*B. micropetala* C.K.Schneid.	India, Manipur	24.67	93.92	N. Macer	04-Jul-05	AS104	Cultivated (J. Harber Coll.)
B_microphylla1	*B. microphylla* G.Forst.	N/A	N/A	N/A	N/A	N/A	1961.063803	Cultivated (RBGE)
B_montana	*B. montana* Gay	Chile : Región X	N/A	N/A	Gardner et al.	15-Jun-05	1993.2827B	Cultivated (RBGE)
B_mucrifolia	*Berberis mucrifolia* Ahrendt	Nepal, Mustang District	28.71194	83.55889	Adhikari, B.	Nov 2009		
B_negeriana	*B. negeriana* Tischler	Chile, Región VIII	N/A	N/A	Hechenleitner Vega	11-Mar-04	200404971	Cultivated (RBGE)
B_nervosa	*B. nervosa* Pursh	Canada, British Columbia	N/A	N/A	Halliwell, Brian	23-Aug-78	1978.2559	Cultivated (RBGE)
B_nevinii	*B. nevinii* A. Gray.	N/A	N/A	N/A	Unknown	Unknown	HC1066	Cultivated (Rancho Santa Ana Botanical Garden)
B_newsppA	*Berberis* new_speciesA	China Yunnan	27.53	99.64	D. E. Boufford et al.	31-Aug-13	43334	
B_newsppB	*Berberis* new_speciesB	China Yunnan	28.57	99.83	D. E. Boufford et al.	31-Aug-13	43304	
B_orthobotrys1	*B. orthobotrys* var. *rubicunda* Ahrendt	Nepal, Rasuwa District	28.21	85.53	Adhikari, B.	03-Aug-07	BL239	
B_orthobotrys2	*B. orthobotrys* var. *rubicunda* Ahrendt	Nepal, Khumbu District	27.79	86.71	N/A	12-Sep-05	DNEP3BY22	
B_pendryi2	*Berberis pendryi* Bh.Adhikari	Nepal, Mustang District	28.81694	83.87	Adhikari, B.	16-Aug-07	EA29	
B_petiolaris1	*B. petiolaris* Wall. ex G. Don	Nepal, Mugu District	29.65	82.11	N/A	12-Jun-08	JRSA122	
B_petiolaris2	*B. petiolaris* Wall. ex G. Don	Nepal, Mugu District	29.65	82.11	N/A	12-Jun-08	JRSA122	Technical Replicate
B_phanera	*B. phanera* C.K. Schneider	China, Sichuan	28.12	101.18	D. E. Boufford et al.	06-Sep-13	43465	
B_polyodonta	*B. polyodonta* Fedde	China Yunnan	N/A	N/A	Lijiang et al.	12-Jun-05	1991.1138	Cultivated (RBGE)
B_praecipua	*B. praecipua* C.K.Schneid.	Bhutan	27.32	89.55	Ruth Liddington	20-Jun-05	AS64	Cultivated (J. Harber Coll.)
B_pruinosa	*B. pruinosa* Franch.	China, Yunnan	27.46	99.9	D. E. Boufford et al.	04-Sep-13	43442	
B_pseudotibetica	*B. pseudotibetica* C. Y. Wu	China, Yunnan	28.29	99.16	D. E. Boufford et al.	19-Aug-13	43134	
B_qiaojianensis	*B. qiaojianensis* S. Y. Bao	China, Yunnan	26.19	103.27	D. E. Boufford et al.	19-Sep-13	43528	
B_spp1	*Berberis* spp.	Nepal, Panchthar District	27.10389	87.9475	Adhikari, B. et al.	08-Jun-07	LKRSB17	
B_temolaica	*Berberis telomaica* Ahrendt	China, Tibet	29.2169	94.21528	A. Clark	NA	AS67	Cultivated (J. Harber Coll.)
B_thomsoniana	*Berberis thomsoniana* C.K.Schneid.	Nepal, Myagdi District	28.40217	83.70247	Adhikari, B.	03-Oct-06	EA101	
B_tibaoshanensis	*B. tibaoshanensis* S. Y. Bao	China, Yunnan	27.61	99.89	D. E. Boufford et al.	04-Sep-13	43436	
B_tsarica1	*Berberis tsarica* Ahrendt	Nepal, Khumbu District	27.94111	86.61	Adhikari, B. et al.	20-Sep-05	DNEP3BY132	
B_wallichiana1	*B. wallichiana* DC.	Nepal, Panchthar District	27.1	87.97	Adhikari, B. et al.	08-Jun-07	LKSRB28	
B_wallichiana2	*B. wallichiana* DC.	Nepal, Rasuwa District	28.17	85.36	Adhikari, B.	02-Aug-07	BL220	
B_wardii	*Berberis wardii* C.K.Schneid	India, Assam	26.00472	94.99806	F. Kingdon-Ward	NA	AS66	Cultivated (J. Harber Coll.)
B_wilsoniae1	*B. wilsoniae* Hemsley	China, Yunnan	27.61	99.72	D. E. Boufford et al.	31-Aug-13	43337	
B_wilsoniae2	*B. wilsoniae* Hemsley	China, Yunnan	24.96	102.66	Z. W Liu	N/A	AS99	Cultivated (J. Harber Coll.)
B_wilsoniae3	*B. wilsoniae* Hemsley	China, Yunnan	29.99	101.95	X. H. Li	05-Jul-05	AS98	Cultivated (J. Harber Coll.)

*Vouchers are deposited at the Herbarium of the Royal Botanic Garden Edinburgh. Missing information is displayed as N/A.*

### Laboratory Work and DNA Sequencing

#### DNA Extraction

DNA was extracted using either the Qiagen DNeasy Plant Kit following the manufacturer’s protocol or the CTAB method (Doyle and Doyle, 1987). The quality of the extractions was checked for the degree of degradation on 1 or 1.5% agarose gels. Furthermore, we performed PCR amplifications of the *rbcL* gene in different dilutions (1:1, 1:10 and 1:100) and finally we measured the DNA concentration on a Qubit^®^ Fluorometer (Life Technologies, Carlsbad, CA, United States), using the dsDNA High Sensitivity kit. The concentrations after extraction ranged from 1.5 to 34.8 ng/μl.

#### Library Preparation and Sequencing

The library preparation for the shotgun sequencing was performed according to Meyer and Kircher (2010). The libraries were sequenced in two runs on a MiSeq^®^ and a NextSeq^®^. Depending on their integrity, the DNA samples were sheared mechanically to a fragment size of approximately 400 bp using a Covaris© sonicator with peak incident power of 75; duty factor of 10%, and 200 cycles per burst. The duration of treatment was chosen according to the observed fragment size on agarose gels and ranged between 30s (medium degradation) and 40s (genomic DNA).

We followed the protocol for blunt-end repair, adapter ligation and adapter fill-in. After each of these steps, the DNA was cleaned-up with AMPure^®^ XP beads (Agencourt^®^). Before the indexing PCR, the DNA quantity was measured on a Qubit©. Depending on the concentration of adapter-ligated libraries, we aimed to use between 50 and 100 ng of DNA as input for the indexing PCR where possible. Higher concentrations may impair the PCR reaction. In order to avoid high duplication levels, a minimal number of PCR cycles were applied. Libraries with concentrations lower than 40 ng were amplified with 16 PCR cycles. If more than 40 ng of library was used for the PCR, 12 cycles were applied. We used the index sequences (“barcodes”) as suggested by the protocol. The final libraries were washed using AMPure^®^ XP beads (Agencourt^®^). We then measured for concentration with Qubit© and assessed the fragment size using Bioanalyzer^®^ (Agilent). The libraries were diluted to 10 mM and pooled together. The libraries were sequenced in two runs on either an Illumina MiSeq^®^ using the MiSeq v2 reagent kit with the 250 bp paired-end option or a NextSeq^®^ with the NextSeq 500 High Output kit performing 150 bp paired-end sequencing.

### Bioinformatics

#### Raw Read Processing and Quality Control

The adapters of the raw reads were removed either with the built-in Illumina software on sequencers or using cutadapt v. 1.10 (Martin, 2011). Raw reads were trimmed using Trimmomatic v.0.33 (Bolger et al., 2014) with the options LEADING:3, TRAILING:3, SLIDINGWINDOW:4:20. Reads from Illumina NextSeq were discarded when shorter than 30 bp and from MiSeq when shorter than 50 bp. The read quality was checked with FastQC (Andrews, 2010).

#### Reference Plastid Genome Reconstructions

The reference genome for *B. aristata7* was reconstructed using a hybrid strategy of read mapping and *de novo* assembly. All reads were mapped to the reference plastid genome of *Berberis bealei* (Ma et al., 2013 GenBank reference KF176554), using the Geneious medium-low sensitivity “Map to Reference” function with five iterations. The resulting contig was then checked manually for low coverage and low pairwise identity regions. One read from each of these regions was extracted and all reads were then mapped against these individual reads as a new reference sequence using the same settings as above. The iterations lead to an extension of the read to a contig (typically up to 2,500 bp). The consensus sequences were then mapped to the reference obtained from the first read mapping. This method allowed large indels in the *B. aristata* reference that were not detected by the read mapping algorithm to be identified. The built-in *de novo* algorithm in Geneious 7.1.7 was used for the *de novo* assembly of the plastid genome. We performed the assembly only with reads that matched to the reference sequence of *B. bealei.* The ten largest contigs, ranging in length from 1,132 to 29,132 bp, were then mapped to the *B. aristata* reference and checked for ambiguities. All reads were then mapped again to the new consensus sequence.

#### Plastid Genome Reconstructions and Alignment

We made our plastid genome reconstructions by mapping to a reference genome, having verified that the levels of variation between B. aristata, our reference, and the chloroplast genome of a member of the distantly related congeneric (B. bealei; Ma et al., 2013 GenBank reference KF176554), were structurally congruent. Reconstructions to a reference permitted a more rapid and cost-effective generation of high quality data than *de novo* assembly. The quality filtered paired-end reads were mapped to a reference genome of *B. aristata7* with Burrows-Wheeler Alignment tool (BWA, ver. 0.7.12, Li and Durbin, 2009). The reference genome was indexed using option “bwa index.” Read pairs that survived the quality check were mapped with default options of the command “bwa mem.” The resulting SAM file was converted to BAM format with “samtools view” and sorted with “samtools sort” in SAMtools v. 1.2 (Li et al., 2009). Optical read duplicates were removed with Picard tools^1^. We used the single nucleotide polymorphism (SNP) calling workflow in GATK (McKenna et al., 2010; Van der Auwera et al., 2013). Regions that contain insertions and deletions are often badly aligned. Therefore, a local realignment process was applied with the command “–T IndelRealigner” in GATK. Variant calling was performed on the realigned BAM files with the “–T HaploTypeCaller” module with haploid settings (“-ploidy 1”). The output is a genomic variant call file (GVCF) that contains base call information for all sites of the markers. The variant calls were then exported with “–T GenotypeGVCFs” to the standard variant call format (VCF). SNP and indel variants were then filtered separately. The first SNP filter applied is quality by depth (QD), which can be considered as the quality of the variant call standardized by the depth of coverage. QD avoids inflation of the Phred quality score for the variant call caused by deep coverage. Variants that had a QD < 2 were filtered out as recommended by Van der Auwera et al. (2013). The FisherStrand (FS) quality filter is a Phred-scaled probability that strand bias exists at a specific site. Specifically, the score is a measure for whether an alternate allele was seen more or less often on either forward or reverse reads. The mapping quality (MQ) in GATK is calculated as the root mean square quality over all reads at a given site. The sites where variance resulted in an MQ score < M 40 were treated as missing data in order to avoid carry-over of reference- specific base pairs. The final sequence was reconstructed with the command “–T FastaAlternateReferenceMaker” in GATK. We checked our pipeline by visual comparison of the final plastid sequence with the BAM file for selected samples.

The plastids were aligned using the MAFFT v7.215 aligner (Katoh and Standley, 2013) with default options. The alignment of repetitive regions such as poly A sequences was not straight-forward, therefore two alignment files were created: the first alignment was used for phylogenetic inference, and blocks where no unambiguous alignment could be constructed were removed. Furthermore, the inverted repeats were removed, since SNP calling on these repeats was difficult to address. Reads with polymorphisms in only one region will map to the other repeat as well. Random mapping to inverted repeat regions often results in apparently heterozygous read alignments, precluding unique assignments of SNPs to a specific inverted repeat. The second alignment was used for the barcoding analysis. Regions were masked (coded as “N”) where no unambiguous alignment was possible.

#### Annotation of Plastid Sequence

The online platforms DOGMA (Wyman et al., 2004) and CpGAVAS (Liu et al., 2012) were used for the annotation of the genome of *B. aristata7.* The full genome sequences were imported into Apollo (Lee et al., 2009). The annotation of *B. aristata* was compared with the previously published annotation of *B. bealei* (Ma et al., 2013). Start and stop codons were checked manually. The annotation was visualized using OGdraw.

#### Universal Barcode Reconstruction

The sequences of *matK, rbcL*, and *trnH-psbA* of *B. aristata* were extracted from the annotated reference *B. aristata7*. The sequences were then aligned to the plastid genomes using BLAT (Kent, 2002). The output was parsed to produce a BED file, which denotes the start and end position of an alignment. The respective sequence was then extracted with the “getfasta” option in BEDTools (Quinlan and Hall, 2010).

A two-step pipeline was devised to reconstruct the ITS2 from shotgun sequencing data. Firstly, reads that map to the ITS2 reference were filtered and then a *de novo* assembly was performed using these reads. Filtering prior to *de novo* assembly reduces computation time substantially. The reference sequence of ITS2 (*Berberis repens*, BOLD accession: HIMS1138-12) was indexed with BWA (Li and Durbin, 2009) using the command “bwa index.” Trimmed and filtered reads were mapped to the reference with “bwa mem.” Mapped reads were then separated from unmapped reads with SAMtools (Li et al., 2009) “samtools view –b –F 4,” resulting in a BAM file with only mapped reads. The mapped reads were then extracted to fastq format using Picard tools (see footnote 1) with the command “SamToFastq.” The reads were then used for *de novo* assembly using SPAdes v3.7.0 (Bankevich et al., 2012) and the longest contig extracted.

#### Barcoding Analysis and Phylogenies

The phylogeny of the plastid alignment was estimated using RAxML v. 8.2.10 (Stamatakis, 2014). The best model of substitution was calculated under the Aikaike Information Criterion in jModeltest2. The ML phylogeny was estimated with 1,000 bootstrap replicates under the GTRGAMMA + I substitution model using the online CIPRES portal (Miller et al., 2010). The whole alignment was considered as a single partition. Members of the compound-leaved *Berberis* were set as outgroup (*B. nervosa, B. polyodonta* and *B. nevinii*).

Potential novel *Berberis-*specific barcodes were explored by extracting SNP positions of the multiple sequence alignment of whole plastid genomes with the program SNP-sites (Page et al., 2016). The SNPs were summarized in 500 bp windows and their distribution plotted with Circos (Krzywinski et al., 2009). Potential barcodes were selected spanning regions where a 500 bp window had a sequence variability of >5%, and a maximum amount of missing/masked data <3%. The 500 bp regions were then compared to the annotated plastid genome and the barcodes were constructed to correspond with genomic regions, such as intergenic spacers that are flanked by conservative regions suitable for primer design. These *Berberis* specific barcodes derived from the whole plastid alignment were evaluated, along with the commonly used barcodes ITS2, *rbcL, matK*, and *trnH-psbA*.

The individual barcode regions were aligned using MAFFT v7.215 (Katoh and Standley, 2013) with default options and were then manually trimmed. A first step was to infer a maximum likelihood tree of the barcode with RAxML v.8.2.9 (Stamatakis, 2014) with 1,000 rapid bootstrap replicates (“–f a”) under the GTRCAT model. The potential barcodes were sorted according to the percent variable sites, percent parsimony informative sites, recovery of *B. aristata* and *B. asiatica* groups and the recovery of groups present in the whole plastid phylogeny. The selected barcodes were concatenated and a maximum likelihood phylogeny was built with the same parameters as described above. Phylogenies of the selected barcodes were inferred under the GTRCAT model in RAxML v. 8.2.9 (Stamatakis, 2014). Additionally, haplotype networks were constructed with the function haploNet in the R package pegas (Paradis, 2010). Finally the alignment of each selected barcode was then reduced to SNP sites only and diagnostic polymorphisms were identified for each group in order to delimit a minimal barcode.

#### Test Data

The first test data consisted of three commercial samples, supposedly of *B. aristata* (Table 2). Sequences for the commercial samples were generated and the sequence data used to make identifications according to the diagnostic loci in Table 4.

**Table 2:** Commercial samples analyzed in this study.

Sample	Form	Company	Place of Purchase
Market 1	Stem/Bark/Root	UK_1	United Kingdom
Market 2	Stem/Bark/Root	UK_1	United Kingdom
Market 3	Powder	India_1	India, Rajasthan (Internet)

*The samples Market1 and Market2 were purchased from the same company. The sample Market3 was purchased from India via the Internet.*

## Results

### Whole Plastid Phylogeny

**FIGURE 1:**
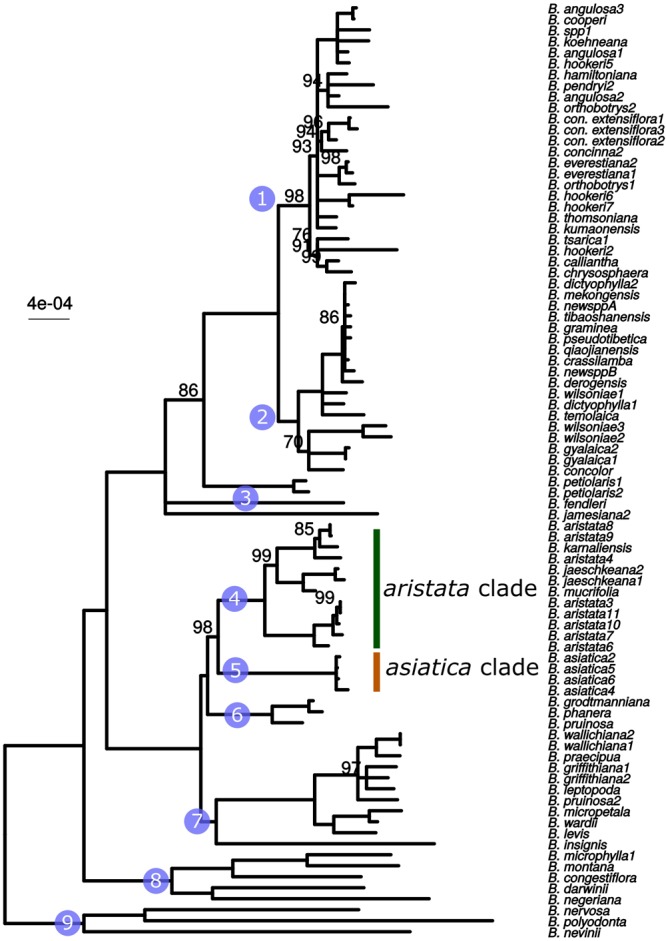
ML phylogeny based on whole plastid sequences. Note that *B. aristata*, in the aristata clade, is a polyphyletic species, but that *B. asiatica* samples in the asiatica group comprise a monophyletic group. Numbers above branches are bootstrap values between 51 and 99. Branches with support <50 were collapsed to polytomies, bootstrap values of 100 are not shown.

The whole plastid phylogeny is shown in Figure 1. Nine groups, eight of which are monophyletic, are identified and numbered 1 to 9. The *aristata, asiatica* and *Mahonia* clades (numbered 4, 5, and 9 in Figure 1) are of most importance in terms of authentication. The plastid phylogeny reveals that *B. aristata* is not monophyletic since *B. jaeschkeana, B. karnaliensis* and *B. mucrifolia* are nested amongst the specimens of this species in clade 4. The topology of the phylogeny is consistent with morphological and biogeographical characters, and with the topology based on nuclear sequence data (Kreuzer et al., in prep.). The annotated plastid sequence of *B_aristata7* is shown in Supplementary Figure S1 and the corresponding sequence is found on Genbank with reference number MK714340.

### Identifying Informative Barcodes

**FIGURE 2:**
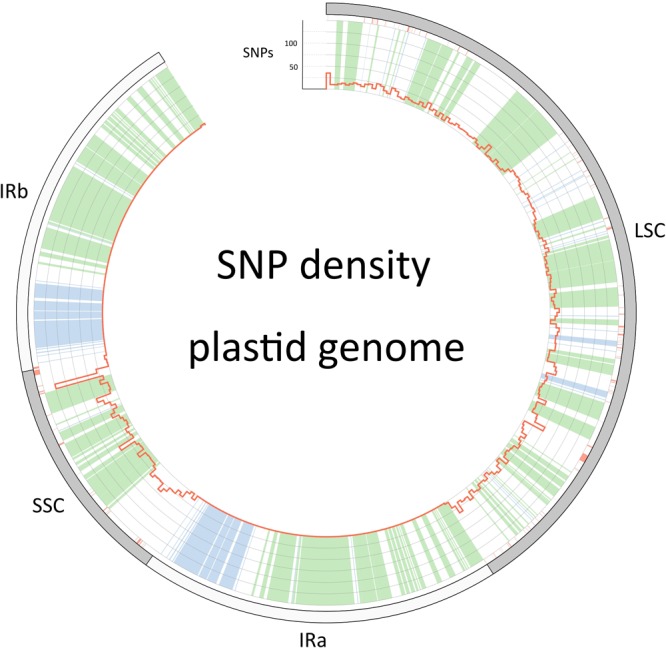
Single nucleotide polymorphism density along the plastid genome (red histograms). The outer circle describes the boundaries of the large single copy, the inverted repeats (IRa and IRb) and the small single copy. Regions that are colored green in the inner circle are coding regions, blue are RNA genes (rRNA and tRNA genes) and white is non-coding sequence. Red color below the outer circle shows regions that have been masked and are thus coded as “N”.

**FIGURE 3:**
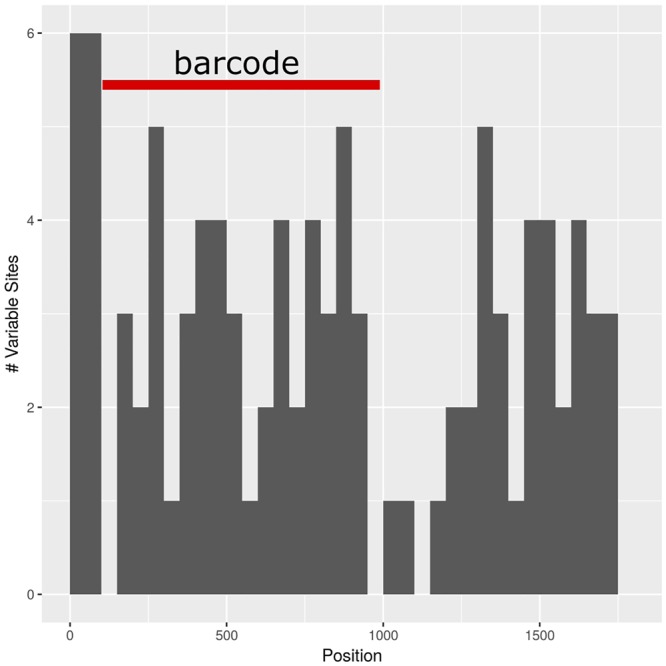
Subselection of barcode regions with the SSC_noncoding2 region. The newly determined barcode is marked in red.

**FIGURE 4:**
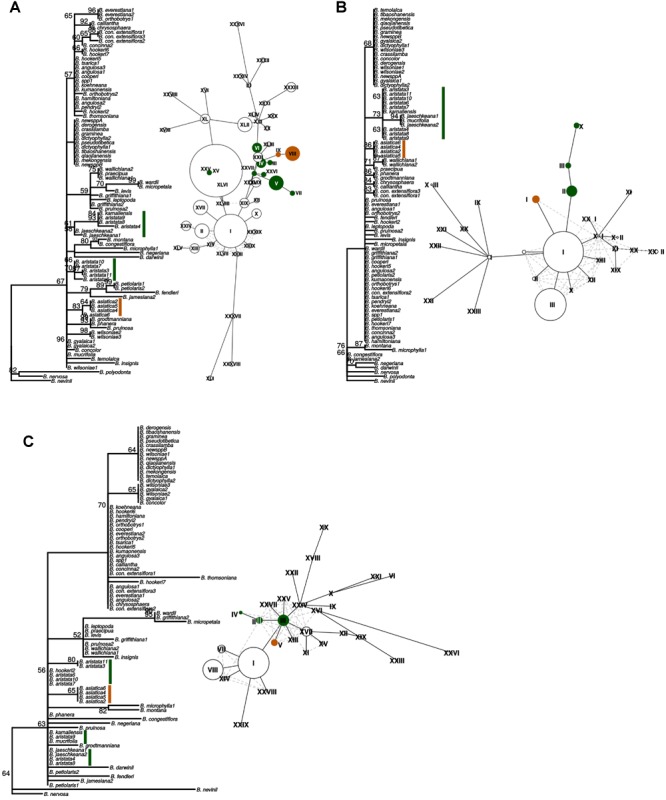
“Maximum likelihood phylogenies and haplotype networks of individual barcodes. **(A)** SSC_noncoding2, **(B)** matK, **(C)** ndhI-ndhG. Values on braches in the phylogeny are ML bootstraps. Species belonging to the *Berberis aristata* clade as recovered from the total plastid phylogeny are identified by green bars, and the *B. asiatica* clades by orange bars on the phylogeny. The same colors are used for the haplotype networks, where Roman numerals indicate different haplotypes and the size of the circles corresponds to the number of samples sharing this haplotype. Species contributing to *B. aristata* clade haplotypes are as follows: SSC_noncoding2 – XXV = *B. mucrifolia* (1 plant), VI = *B. aristata* (2 plants), IV = *B. aristata* (2 plants), III = *B. aristata* (1 plant), XXVI = *B. jaeschkeana* (1 plant), XXVII = *B. jaeschkeana* (1 plant), V = *B. aristata* (2 plants) and *B. karnaliensis* (1 plant), VII = *B. aristata* (1 plant); matK – X = *B. jaeschkeana* (2 plants) and *B. mucrifolia* (1 plant), III = *B. aristata* (3 plants) II = *B. aristata* (5 plants) and *B. karnaliensis* (1 plant); ndhI-ndhG – IV = *B. aristata* (2 plants), II = *B. aristata* (2 plants) and *B. hookeri* (1 plant), III = *B. aristata* (3 plants) and *B. jaeschkeana* (2 plants) and *B. karnaliensis* (1 plant) and *B. mucrifolia* (1 plant).”

The barcoding analysis aimed to find a set of informative nucleotides that are unique to clades of interest. The topology of the whole plastid genome phylogeny was used to determine evolutionarily meaningful groups, termed Operational Phylogenetic Units (OPUs). Barcodes were then constructed for identifying these OPUs, rather than individual species. A barcoding method based on diagnostic characters was preferred over distance or purely phylogenetic approaches, because of its ease of application to regulatory purposes and to provide an alternative approach in an evolutionarily complex group. The density of SNPs in 500 bp windows along the whole plastid alignment is shown in Figure 2. The bins contained between 0 and 124 variable sites per 500 bp. The inspection of bins with >25 SNPs (5%) resulted in 21 potential barcode regions. Several of the highly variable bins fell into regions where the alignment was partly masked due to ambiguous alignment, leaving 13 bins for further inspection. Two neighboring bins were combined into a single potential barcode of 1,000 bp, and a set of four bins combined into a 2000 base pair barcode. The barcode of 2,000 bp (SSC_noncoding2) was further examined by partitioning the alignment into 50 bp windows and reducing the barcode size (SSC_noncoding2, Figure 3). The *trnH-psbA* intergenic spacer was identified among one of the seven highly variable regions, and together with the *matK, rbcL* and ITS2 barcodes, selected because they are commonly used barcode regions, eleven barcode candidates were investigated (Table 3). None of the individual barcodes retrieved phylogenies with the same topology as the whole plastid phylogeny. Although the *matK* phylogeny is not well resolved overall, species from the *aristata* and *asiatica* groups were recovered. *B. asiatica* is monophyletic in the non-coding SSC_noncoding2 phylogeny, but species from the *aristata* clade are separated into two groups. The percent variable sites varied between 2.2 in *rbcL* and 9.85 in the intergenic spacer *ndhI-ndhG* (Table 3) and the latter was chosen along with *matK* and SSC_noncoding2 as barcodes for phylogenetic and haplotype analysis (Figure 4).

**Table 3:** Barcode selection resulting from investigating variability patterns across whole plastid alignment.

Barcode	Length (bp)	Var	% Var	PIS	% PIS	*aristata* recovered	*asiatica* recovered
ITS2 (nuclear)	560	45	8.04	24	4.29	No	Yes
***matK***	**1530**	**39**	**2.55**	**18**	**1.18**	**Yes**	**Yes**
*ndhF* (partial)	802	40	4.99	23	2.87	No	Yes
***ndhI-ndhG***	**501**	**48**	**9.58**	**18**	**3.59**	**No**	**Yes**
*rbcL*	1452	32	2.20	21	1.45	No	Yes
*rbcL-atpB*	770	32	4.16	19	2.47	No	Yes
*rbcL-psaI*	626	59	9.42	28	4.47	No	Yes
*rpl32-ndhF*	1119	80	7.15	40	3.57	Partly	Yes
SSC_noncoding1	741	52	7.02	29	3.91	Partly	No
**SSC_noncoding2**	**790**	**46**	**5.82**	**27**	**3.42**	**Yes**	**Yes**
*trnH-psbA*	580	43	7.41	24	4.14	No	Yes

*matK and *rbcL* were not identified as highly variable but included in the study. Var = Variable sites; PIS = parsimony informative sites; “*aristata* recovered” and “*asiatica* recovered” indicates whether the clades were recovered in the respective phylogeny. Barcode selection resulting from investigating variability patterns across whole plastid alignment. The DNA barcodes that were selected are highlighted in bold font.*

**FIGURE 5:**
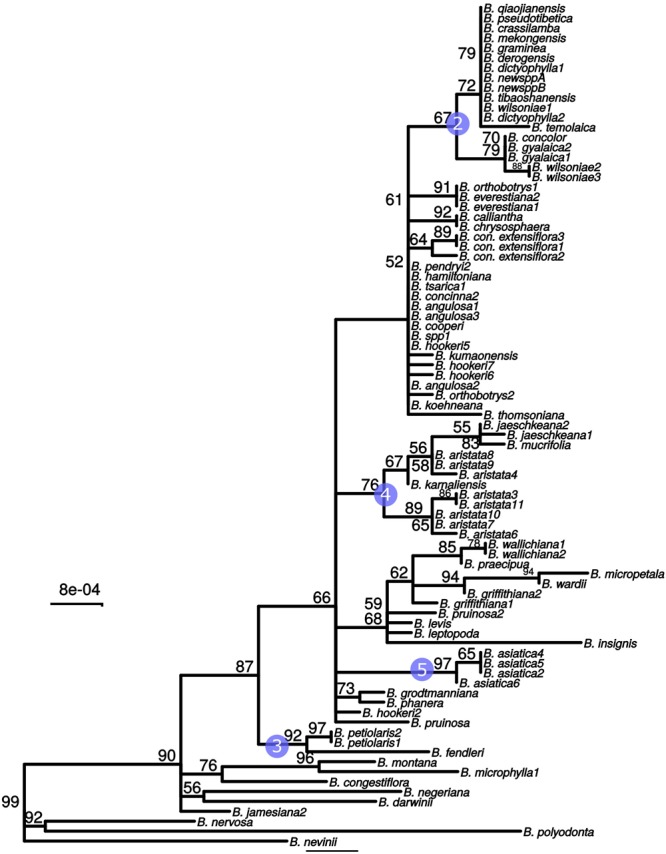
Maximum likelihood tree from the concatenated barcodes *matK*, SSC_noncoding2 and *ndhI-ndhG*. Nodes with bootstrap support <50 were collapsed to polytomies. Bootstrap values between 50 and 99 are shown above branches. No number indicates a bootstrap value of 100. Numbered circles indicate groups that were recovered in the whole plastid phylogeny (see Figure 1).

These three barcodes yielded 133 variable positions in total. Nine positions were sufficient to identify seven of the nine groups with clade-specific nucleotide variants. Groups 3 and 8 (Figure 1) share a barcode, in other words their barcodes are identical. The phylogeny of the concatenated barcodes *matK*, SSC_noncoding2 and *ndhI-ndhG* barcodes is shown in Figure 5. The topology of the tree differs substantially from the total-evidence tree inferred from whole plastid sequences. However, four of the major clades are identified in both trees. Haplotype networks constructed for each of the separate data sets showed variation in the haplotype associated with the *B. aristata* clade (Figure 4). There was no haplotype unique to *B. aristata:* for the SSC_noncoding2 region one of the *B. aristata* haplotypes is found also in *B. karnaliensis;* for the matK region there is also a haplotype shared between *B. aristata* and *B. karnaliensis*; for ndhI-ndhG there is a haplotype found in *B. aristata, B. jaeschkeana, B. karnaliensis* and *B. mucrifolia.* The lack of species-specific haplotypes even in these most variable regions underlines the necessity of a clade-based approach. However, for pharmacopoeial purposes the haplotype networks reveal separation of the *B. aristata* clade haplotypes and *B. asiatica* haplotypes.

### Testing Barcodes

The minimal barcode consists of nine positions and includes barcodes unique to seven groups. No unique SNPs were identified for groups 3, 6, and 8. No individual barcode for groups 6 and 8 could be constructed (Table 4). The barcodes were evaluated with the test data set. The commercial samples Market1 and Market2 were identified as belonging to the *Mahonia* clade. The sample Market11 shared the barcode with *B. asiatica* samples.

**Table 4:** *Top*: Matrix of informative barcode positions.

	*matK*				*ndhI-ndhG*			SSC_noncoding2	
Position (bp)	755	857	976	1428	151	182	326	47	700
**clade. 1**	AA	GG	GG	GG	CC	AA	CC	AA	GG
**clade. 2**	AA	GG	GG	AA	CC	AA	CC	AA	AA
**clade. 3**	AA	GG	GG	GG	AA	AA	CC	AA	AA
***aristata –* clade (4)**	CC	AA	GG	GG	CC	AA	CC	AA	AA
***asiatica –* clade (5)**	AA	GG	GG	GG	CC	CC	CC	AA	AA
**clade. 6**	AA	GG	GG	GG	CC	AA	CC	AA	AA
**clade. 7**	AA	GG	AA	GG	CC	AA	CC	AA	AA
**SA clade (8)**	AA	GG	GG	GG	AA	AA	CC	AA	AA
***Mahonia –* clade (9)**	AA	GG	GG	GG	AA	AA	AA	CC	AA
	**Test Samples**								
**Market1**	AA	GG	GG	GG	AA	AA	NN	CC	AA
**Market2**	AA	GG	GG	GG	AA	AA	NN	CC	AA
**Market3**	AA	GG	GG	GG	CC	CC	CC	AA	AA

*The positions are relative to the consensus of the multiple sequence alignments of each barcode. “SA clade” stands for South American clade. *Bottom*: Results of the test samples. Market1, Market2, and Market3 are commercial samples. and Mixture1 and Mixture2 are *in silico* mixtures. Numbers below multiple base calls represent the ratio of nucleotides in the mapping.*

## Discussion

DNA barcoding for quality assurance and pharmacovigilance has great potential and is likely to be implemented as a routine diagnostic method. In this study, we present an approach for barcoding of an evolutionarily complex group of species and demonstrate that these barcodes can identify the species in commercial samples. Our purpose was to provide a barcode for pharmacopoeial purposes that discriminates *B. aristata* and *B. asiatica* since these are the pharmacopoeial species and the main substitute, respectively. We present a solution for barcoding that meets regulatory needs.

With the emergence of new sequencing technologies, whole plastid sequencing has been proposed as an extension of the current barcoding concept (Coissac et al., 2016). It has been shown that whole plastid sequences increase phylogenetic resolution (Parks et al., 2009) and simultaneously increase the effectiveness of discriminating between species. In this study, we show how whole plastid next-generation sequencing can be used to investigate sequence variability patterns for the discovery of informative DNA barcodes. We confirm the difficulty of barcoding *Berberis* species as suggested by Roy et al. (2010), even when whole plastid sequences are used for comparison. Although the sampling was limited, with only a few of the species represented with multiple samples, the low resolution of the plastid phylogeny at shallow phylogenetic levels and the presence of polyphyletic species (e.g., *B. aristata*) indicates evolutionary reasons for the failure of barcoding this genus to species level (Mutanen et al., 2016). DNA barcoding is challenging in groups where frequent hybridization occurs in conjunction with plastid capture or where lineage sorting has not yet been completed (Fazekas et al., 2009). A salient point arising from our study is that the pharmacopoeial species, *B. aristata*, is polyphyletic. One explanation for this finding is hybridization, a phenomenon documented in *Berberis* (Adhikari et al., 2012). Low resolution among the closely related species of *Berberis* as reported in the whole plastid phylogeny, could point toward retention of ancestral polymorphism or incomplete lineage sorting (Naciri and Linder, 2015). Misidentification of *B. jaeschkeana, B. karnaliensis* and/or *B. mucrifolia* is unlikely, since these have been included in recent revisionary work (Adhikari et al., 2012). Polyphyletic species are likely to persist where they are morphologically robust entities, and the development of methods for their identification, in this case for pharmacopoeia, benefits from understanding of their evolutionary history. The case of barcoding medicinal *Berberis* species provides an example of how barcoding for regulatory purposes in an evolutionarily complex group can be approached. Phylogenies can be essential for formulating adequate barcoding hypotheses; the whole plastid phylogeny reveals that at least three species are nested in the clade with the main species. The polyphyly of *B. aristata* indicates that universal barcodes are unlikely to delineate these species, and haplotype analysis shows this is the case for three of the most variable regions. Furthermore, several clades show low resolution at terminal branches. We have therefore adapted our classification scheme and defined meaningful OPUs that do not correspond to existing species limits. OPUs are the entities that can be discriminated by the barcodes put forward. The OPUs in this study are delimited using an integrative approach based on the interpretation of a whole plastid phylogeny, coupled with the detection of diagnostic nucleotides in relatively short barcodes for well-supported groups. These DNA barcodes can be targeted by PCR and Sanger sequencing and therefore offer a simple and fast identification test for regulatory purposes and quality control. Appropriate OPUs would be identified on a case-by-case basis for other evolutionarily complex groups for regulatory purposes. This is because for evolutionarily complex groups barcodes do not confirm species identity. The novelty of our approach lies in using whole plastid phylogeny to identify of short, easily amplified markers that incorporate clade-specific SNPs, and although we expect it to be more widely applicable it is only appropriate when the non-pharmacopoeial species belonging to the OPU are neither candidate adulterants nor substitute species, as is the case here.

The barcode presented in this study is based on diagnostic nucleotides for groups of species, referred to here as OPUs. Like the morphological classification of species, diagnostic methods provide a set of unique characters to assign specimens to species or species groups (Little and Stevenson, 2007). Diagnostic methods are particularly well-suited to pharmacopoeial purposes because a sequence generated from test material can be compared to a published sequence in a way that is comparable to other pharmacopoeial standards. The barcode we propose would require the user to amplify and sequence three regions, whereas the barcodes included in the British Pharmacopoeia to date are single regions (British Pharmacopoeia, 2016). We have limited the number of loci that would be part of the test to three because incorporating more loci would make the test more unwieldy for users. Limiting the number of regions necessarily reduces the number of informative sites. Identifying the most informative regions, as we do here, is therefore important. A deficiency of the diagnostic method is that further samples might show variation that is not present amongst the samples used for barcode design. However, there is scope to modify the published barcodes, perhaps by using the IUPAC nucleotide codes, if novel variants are reported.

The diagnostic method has been implemented in various analysis tools (Sarkar et al., 2008; Weitschek et al., 2013), mainly for specimen identification. Some of the algorithms use logic mining techniques (Bertolazzi et al., 2009). Logic mining for DNA barcoding refers to a two-step process, in which the barcode is first reduced to a set of very informative nucleotides and thereafter a logic mining method is applied, to define a set of formulas for separating the species. More recent approaches, such as BLOG 2.0 (Weitschek et al., 2013), provide a diagnostic, character-based methodology to species identification that is based on supervised machine learning. Character-based approaches circumvents analytical issues such as the nearest-neighbor problem in distance-based methods (DeSalle et al., 2005). Although the *in silico* mixtures presented in this study were created from the samples that were used for producing the DNA barcode and are therefore not true test samples, the analysis demonstrates the utility of analyzing mixed samples based on diagnostic nucleotides when shotgun sequencing data is available.

We believe that the development of clade-specific DNA barcodes is the way forward when investigating evolutionarily complex species. The barcodes we present are readily understandable and easily applicable for large-scale and routine testing of samples using PCR and Sanger sequencing. DNA barcoding is beyond doubt a powerful method for specimen identification, but its implementation as a routine process for quality assurance (Sgamma et al., 2017) and pharmacovigilance (de Boer et al., 2015) will depend on the ease of application. Neither phylogenetic nor distance methods are appropriate, since they depend on large databases, sophisticated tools and lack objective criteria. For this reason, the British Pharmacopoeia (BP) approach is to present a sequence which samples must match for authentication. Pharmacopoeias ensure the safe use of pharmaceuticals by defining certain quality standards and DNA barcodes have recently been published in the BP for the first time (British Pharmacopoeia Commission, 2017). The question “does this sample correspond to the pharmacopoeial species?” is addressed by comparison to the pharmacopoeial sequence, since methods based on diagnostic nucleotides provide an easy and straight-forward way to answer the question. Identifying such sequences for inclusion in a pharmacopeia is the challenge addressed by this study. The whole plastid approach described here could become a model that can be applied to species that are difficult to resolve. Success depends on devising a sampling strategy that includes species that are closely related to the target species. Furthermore, the inclusion of distantly related, congeneric species increases the confidence in detected diagnostic nucleotide polymorphisms.

## Author Contributions

JH, CH, CP, and MK contributed to the conception and design of the study. BA and CP provided samples and made taxonomic identifications. CH and MK conducted the laboratory work. MK performed the data analysis and wrote the first draft of the manuscript. All authors contributed to manuscript revision, read and approved the submitted version.

## Conflict of Interest Statement

The authors declare that the research was conducted in the absence of any commercial or financial relationships that could be construed as a potential conflict of interest.

## Footnotes

1 http://broadinstitute.github.io/picard, last accessed June 30, 2017

## Supplementary Material

FIGURE S1 Gene map of the plastid genome of *Berberis aristata*. Genes on the outside of the circle are transcribed clockwise and genes on the inside anti-clockwise. The dark gray histograms in the inner circle show the GC content.
