# Supplementary figures and images for: Phylogenomic Approaches to DNA Barcoding of Herbal Medicines: Developing Clade-Specific Diagnostic Characters for Berberis
Source: Front Plant Sci. 2019 May 14;10:586. doi: 10.3389/fpls.2019.00586 (PMC6527895; doi:10.3389/fpls.2019.00586)

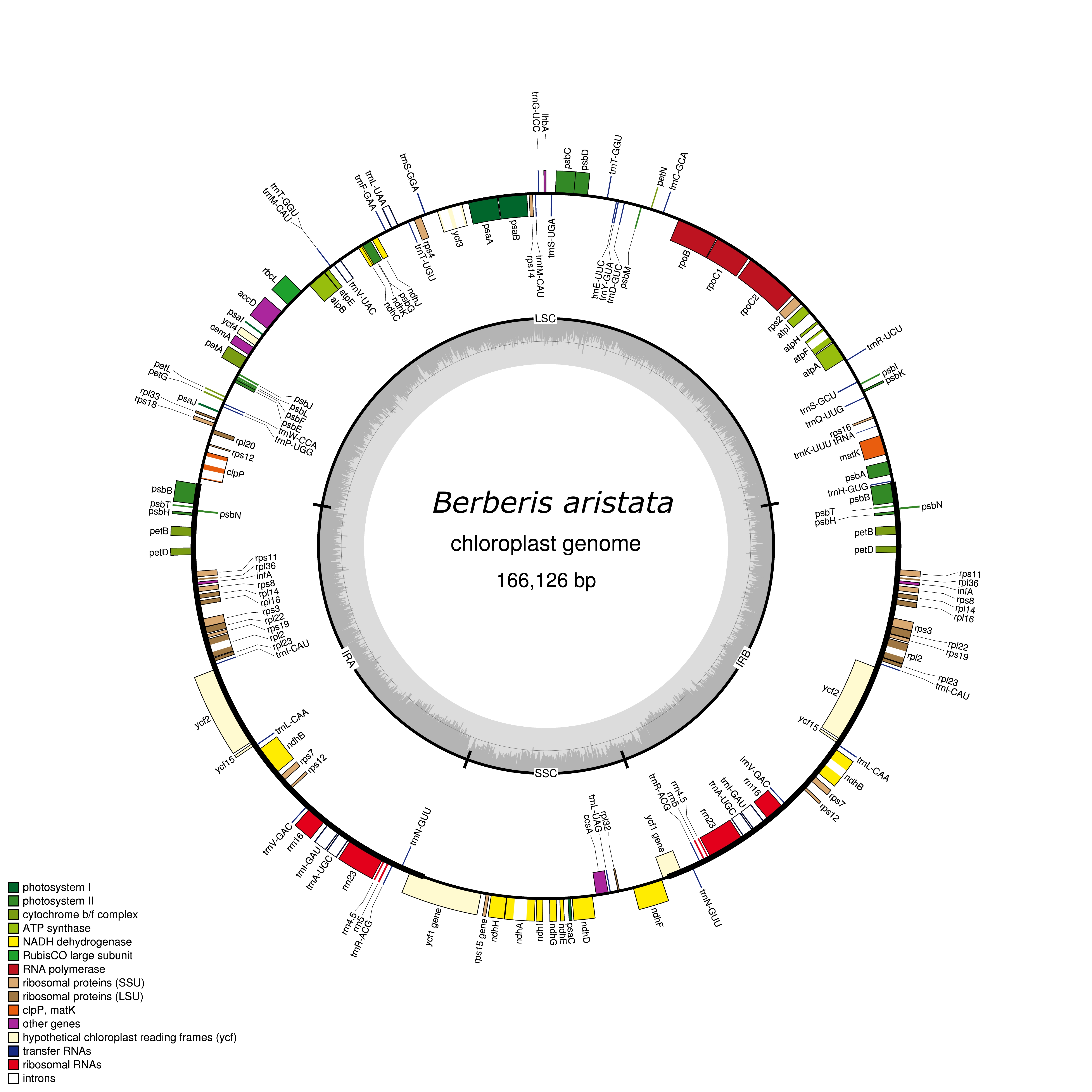

Supplement: FIGURE S1 — Gene map of the plastid genome of Berberis aristata. Genes on the outside of the circle are transcribed clockwise and genes on the inside anti-clockwise. The dark gray histograms in the inner circle show the GC content. [file Image_1.JPEG]
